# On the road again: Impact of travel distance on outcomes for lung cancer

**DOI:** 10.1016/j.xjon.2023.05.002

**Published:** 2023-05-27

**Authors:** Michael A. Eisenberg, Mara B. Antonoff

**Affiliations:** Department of Thoracic and Cardiovascular Surgery, University of Texas MD Anderson Cancer Center, Houston, Tex

To the Editor:

**Figure:**
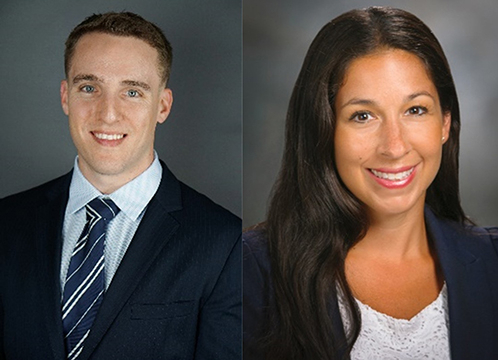

The authors reported no conflicts of interest.

The *Journal* policy requires editors and reviewers to disclose conflicts of interest and to decline handling or reviewing manuscripts for which they may have a conflict of interest. The editors and reviewers of this article have no conflicts of interest.

We congratulate Logan and colleagues^1^ on their excellent article describing their investigation of travel distance influence on the receipt of adjuvant chemotherapy for patients with stage II-IIIA non–small cell lung cancer (NSCLC)^1^ within the National Cancer Database (NCDB). Following the promising results of the Impower010 and ADAURA trials, which supported the expanded use of adjuvant immunotherapy^2^ and targeted therapy,^3^ identifying and mitigating potential barriers to receipt of indicated adjuvant therapy is particularly timely. This study is the largest to date evaluating the role of travel distance as a potential barrier to receipt of postoperative therapy, providing evidence of suboptimal patient outcomes for those who fail to receive the full complement of guideline-concordant care.

The authors importantly found that with the regionalization of high-volume centers for treatment of NSCLC, those patients who failed to receive adjuvant chemotherapy traveled greater distances than those who received indicated adjuvant treatment (13 vs 11.2 miles, *P* < .001).^1^ Moreover, they further showed that the benefits of treatment at high-volume centers were diminished when adjuvant care was fragmented as a result of distance (*P* < .001).^1^ The deleterious effects of travel distance on receipt of adjuvant treatment demonstrated by this study highlight the potential utility of telehealth as a bridge to delivery of care to patients residing farther from operative centers.

The findings Logan and colleagues^1^ are concordant with our single-institution evaluation of travel distance as a barrier to receipt of indicated adjuvant therapy in pathologic stage II-III NSCLC.^4^ Within our cohort, travel distance was similarly shown to have a detrimental impact on the likelihood of undergoing appropriate postoperative therapy. We selected a threshold of travel >100 miles based on previous work by Bostock and colleagues,^5^ which revealed the negative impact of travel distance on adherence to recommended postoperative surveillance imaging in NSCLC, and found that traveling >100 miles also led to substantially reduced likelihood of receiving indicated adjuvant surveillance (odds ratio, 0.13; 95% confidence interval, 0.06-0.26, *P* < .001).^4^

Again, the authors should be commended for their thoroughness in the evaluation of operative center volume and distance from patients' homes. It should be noted, however, that a limitation of this study was the use of the NCDB, which calculates travel distance “as the bird flies,” as opposed to using mapped roads, which may lead to a degree of underreporting of distance. Moreover, as the NCDB is hospital-centric as opposed to patient-centric, there may be a degree of underreporting, particularly in rural patients, who may not receive their adjuvant care at a NCDB-accredited hospital. These issues were acknowledged by the authors, and despite these inherent limitations, the authors should be applauded for their efforts to improve access to care to all patients.

Ultimately, the investigators of this study were able to demonstrate undeniable barriers to receipt of adjuvant care for patients who underwent resection for stage II-IIIA NSCLC, which is a critical step as we aim to provide guideline-directed therapy to *all* patients. It is clear that identifying and addressing the specific social, economic, and cultural components of these travel barriers will provide an actionable opportunity to mitigate disparities in outcomes for patients with lung cancer.
